# Short versus longer duration antibiotic treatment for urinary tract infections in companion animals: a systematic review and meta-analysis

**DOI:** 10.1186/s12917-025-04722-y

**Published:** 2025-04-17

**Authors:** Fiona Emdin, Sean W. X. Ong, Clare McGall, Valerie Leung, Kevin L. Schwartz, Bradley J. Langford, Kevin A. Brown, Susan Massarella, Nick Daneman

**Affiliations:** 1 Public Health Ontario, Toronto, ON Canada; 2 Institute of Health Policy, Management and Evaluation, University of Toronto, Toronto, ON Canada; 3 Division of Infectious Diseases, Sunnybrook Health Sciences Centre, Toronto, ON Canada; 4 Global Strategy Lab, York University, Toronto, ON Canada; 5 Li Ka Shing Knowledge Institute, Unity Health Toronto, Toronto, ON Canada; 6 Dalla Lana School of Public Health, University of Toronto, Toronto, ON Canada; 7 Department of Infectious Diseases, at the Peter Doherty Institute for Infection and Immunity, University of Melbourne, Melbourne, VIC Australia

**Keywords:** Antibiotics, Antimicrobial stewardship, Decision making, Veterinarians, Cats, Dogs, Urine culture

## Abstract

**Background:**

Unnecessarily prolonged antibiotic durations may contribute to the development of resistance in both humans and animals. Veterinarians need evidence supporting antibiotic treatment durations. This systematic review and meta-analysis aimed to compare the efficacy of shorter durations of antibiotic treatment to longer durations in treating urinary tract infections (UTIs) in dogs and cats.

**Methods:**

Four databases (MEDLINE, Scopus, EMBASE, and CAB Abstracts) were searched from inception to October 2nd, 2024. Studies that reported the impact of antibiotic treatments of different durations for simple UTIs in dogs or cats and reported a primary outcome of interest, specifically clinical or microbiological resolution of the UTIs, were included. For each study, two reviewers independently screened extracted data and evaluated the risk of bias. Random effects models were used to compare pooled risk ratios of cure rates.

**Results:**

Of 2,324 studies screened, we identified three studies (two randomized and one nonrandomized controlled trial) which met our inclusion criteria for meta-analysis. Studies examined only 26 animals (9 events) across their short-duration arms and 28 animals (17 events) across long-duration arms. All studies were assessed as having high or serious risk of bias. The pooled risk ratio for cure with short versus longer durations of treatment was 0.55, 95% CI: 0.23–1.27; the evidence was graded as very low certainty. Studies compared 1 to 3-day durations, 3 days to 14-day and 3 days to 21-day durations.

**Conclusion:**

Based on this data alone, we cannot make conclusions about the efficacy of short compared to long antibiotic durations for treating UTIs in cats and dogs; due to the low numbers of included studies and patients, the confidence intervals for the pooled risk ratio were wide and could be consistent with inferiority or superiority of shorter treatment. Existing evidence supports shorter durations of antibiotics for treating sporadic UTIs in dogs and cats, however this systematic review and meta-analysis highlights that this is still a serious knowledge gap that must be addressed. Studies that examine optimal antibiotic durations for treating UTIs in dogs and cats are urgently needed to support clinical decision-making, inform guidelines, and improve antimicrobial stewardship in veterinary medicine.

**Systematic review registration:**

Open science framework (10.17605/OSF.IO/2YJPM).

**Supplementary Information:**

The online version contains supplementary material available at 10.1186/s12917-025-04722-y.

## Introduction

Antimicrobial resistance (AMR) is a serious public health threat. In 2021, it was estimated that AMR contributed to the deaths of almost 5 million people [1]. Antimicrobial use in animals is an important driver of AMR in humans [2, 3] which is why a One Health approach, meaning coordinated action across human, animal, and environmental sectors, is needed to address the growing threat of AMR [4].

Veterinarians need evidence and clinical guidelines to support their prescribing practices and reduce areas of potential misuse and overuse of antimicrobials like antibiotics [5,6]. In particular, in companion animal species, like cats and dogs, there is a need for additional research supporting antibiotic treatment regimens [7]. Not only will longer-duration antibiotic treatments potentially drive the development of resistant infections in animals in the future [8], but animals are also at higher risk of developing side effects with longer courses of antibiotics [9]. Additionally, longer treatment durations cost owners more and may result in reduced owner compliance [10]. Even for diseases for which antibiotics are commonly prescribed, such as urinary tract infections (UTIs) [11, 12] guidance is often based on human studies, and there is limited dog and cat-specific evidence available [13].

UTIs are the most common infectious disease in dogs, affecting 14% of dogs over their lifetime [14]. They are also one of the most common presenting issues resulting in an antibiotic prescription in both dogs and cats [11]. The International Society for Companion Animal Infectious Disease (ISCAID) recently updated its guidelines on treating UTIs in dogs and cats [15]. In the case of sporadic bacterial cystitis or simple UTIs, these guidelines recommend 3 to 5 days of treatment with amoxicillin or trimethoprim-sulfonamide. Prior to this update, ISCAID guidelines recommended longer durations of treatment for 7 days, and before 2011, durations of 10 to 14 days were recommended [16]. Although shorter durations of therapy are now recommended, these newly updated guidelines specifically highlight the lack of veterinary evidence supporting duration recommendations [15]. A 2021 study examining antibiotic prescriptions for dogs with suspect UTIs found that while durations had decreased in 2018 compared to 2016 and 2017, the median prescribed duration was still 10 days in 2018 [17]. Only one systematic review from 2015 has examined antibiotic efficacy and duration of treatment in UTIs in dogs [18].

To fill this knowledge gap, support clinical guideline adoption and guide veterinary prescribing practices, we conducted a systematic review and meta-analysis to answer the question: are shorter durations of antibiotic treatment as effective in treating simple urinary tract infections (as measured by clinical or microbiological cure) in dogs and cats when compared to longer antibiotic therapy duration?

## Methods

### Protocol and registration

This protocol was registered with Open Science Framework (10.17605/OSF.IO/2YJPM*)* and was developed in line with the Preferred Reporting Items for Systematic Review and Meta-Analysis (PRISMA) guidelines. A populated PRISMA checklist is available as an Appendix (*Appendix 1*).

### Inclusion and exclusion criteria

A full list of inclusion and exclusion criteria can be found in Table 1.

**Table 1:** Summary of inclusion and exclusion criteria

Criteria	Inclusion criteria	Exclusion criteria
The study assesses the population (P) and disease of interest.	We only included studies which assessed experimentally induced (which refers to introducing bacteria to establish a UTI in a healthy laboratory animal) or sporadic bacterial cystitis (also called simple UTIs) in dogs and cats, meaning animals must be healthy. No restrictions on breed or age of animals were applied.	Studies that considered other animal species or humans were excluded. Studies in unhealthy dogs or cats (animals with underlying anatomic, functional or systemic diseases which might predispose them to UTIs) or those with recurrent bacterial cystitis (meaning 3 or more sporadic bacterial cystitis events in the past 12 months or two or more events in the past six months^15^), prostatitis or pyelonephritis were also excluded.
The study compares (C) the impact of treatment (I).	To be included the study must have compared the impact of antibiotic treatment durations (and report type of antibiotic, dose and duration of treatment) on spontaneous or induced urinary tract infections. Studies also must have compared the same antibiotic and drug for both arms/comparison groups. Studies could compare any antibiotic durations; to prevent exclusion of relevant studies (i.e., those which might compare 7-to-10-day durations) we compared efficacy of antibiotic durations continuously across a 1 day to > 14-day duration range. Additionally, studies examining any antibiotic type and any routes of administration (PO, IM, IV or SQ) were eligible for inclusion.	If the study reported on the impact of antibiotic treatment on a different disease such as prostatitis, or pyelonephritis it was excluded. Studies which reported on use of antibiotics for prophylaxis of UTI were excluded. Studies which compared different antibiotics in each arms/comparison group were excluded.
The study assesses a primary outcome (O) +/- secondary measures of interest.	Studies needed to report on the primary outcome of interest: clinical or microbiological resolution of a sporadic or induced urinary tract infections in dogs or cats 1 to 14 days after treatment as defined by authors. Secondary outcomes included long term (14 to > 30 day) clinical or microbiological cure rates, mortality and any adverse events reported.	If the study did not report on clinical or microbiological resolution of urinary tract infections, it was excluded.
The study design meets methodological requirements [19].	To be included studies had to be peer-reviewed. Due to an anticipated low number of RCTs performed on this topic, observational studies such as nonrandomized controlled trials, case-control or cohort studies were also included. We also opted to include published abstracts.	Qualitative study designs, editorials, reviews, commentaries, case series, case reports, pre-prints, study protocols, dissertations, and posters were excluded.

### Information sources

We searched the following databases to identify evidence published in scientific journals: MEDLINE (Ovid platform), Embase (Ovid platform), CAB Abstracts (Ovid platform), and Scopus (Elsevier platform). These databases were searched from inception to October 2nd, 2024; no publication date limits or limits on the language of publication were applied. Non-English studies were translated using Google Translate and included in the screening.

### Search strategy

For each database, the search strategy was designed to retrieve records containing at least one search term (in major topic heading, title keyword, or natural language descriptor fields) related to the concept of antibiotics, urinary tract infections, domesticated cats or dogs and duration or treatment course. To identify relevant evidence on this topic, Public Health Ontario (PHO)’s Library Services [20] designed and executed our scientific literature searches. A copy of the full search strategy for each database can be found in *Appendix 2*.

### Study selection

Title, abstract, and full-text screening were completed independently by two reviewers (FE, SO, ND, CM) in the systematic review software Covidence [21]. Any disagreements were resolved through consensus. We also hand-searched the cited references of all studies included in this review to identify any additional studies.

### Data management and collection

Two reviewers independently extracted data for all included studies into a tailored Excel extraction form (FE, CM), which was tested first on two studies. Any conflicts that were identified were resolved through consensus. Researchers extracted general information on studies (author name and year of publication), study population parameters (species, number of animals, age/sex and health status of animals), disease information (induced or sporadic bacterial UTI, urine sampling method), antibiotic information (drug, dose, duration), primary outcomes (effect measure, evaluation time, evaluation method), and secondary outcomes (long term cure rates/measures, mortality, adverse events) if reported. If not reported, the raw data to calculate these effect measures were extracted instead. A full list of data items that were extracted can be found in *Appendix 3*.

### Missing data

Missing data was recorded on extraction forms. Authors of included studies were contacted twice to provide missing data using corresponding author emails or public emails found by searching the web; studies were excluded in the event of author nonresponse if meta-analysis could not be performed with the available data.

### Risk of bias

Risk of bias was assessed for included studies using two risk of bias tools: the Cochrane Risk of Bias 2 tool for randomized trials [22], which assesses studies as having high, low or some concerns for bias across five domains, and ROBINS-I [23] for non-randomized studies of interventions which assess studies as having low, moderate, serious or critical risk of bias across seven domains. The risk of bias was assessed by two reviewers (FE, CM) concurrently with data extraction, and consensus was used to resolve any disagreements.

### Outcomes

To be included, studies needed to report on the primary outcome of interest: clinical or microbiological resolution of sporadic or induced urinary tract infections in dogs or cats after treatment as defined by authors. We assessed the efficacy of antibiotic durations continuously across a 1-day to > 14-day duration range. Examples of how authors might define the clinical resolution of symptoms are the resolution of polyuria, pollakiuria, haematuria, stranguria and/or dysuria, while microbiological cure was commonly defined as a negative aerobic bacterial urine culture or culture that yielded < 10^3^ CFU/mL of bacteria. Secondary outcomes included long-term (14 to > 30 days) clinical or microbiological cure rates, mortality and any adverse events reported.

To be included the study must have compared the same antibiotic for both arms/comparison groups. We opted not to include studies which compareddifferent antibiotics in each arms/comparison group [24, 25] as we felt this might introduce variability which would make it difficult to directly compare outcomes and prevent pooling the results in a meaningful way (e.g. if we saw higher rates of side effects in shorter or longer arms it might be due to different side-effect profiles between the two antibiotics).

### Summary effect measures for outcomes

We collected either (i) effect measures for both primary and secondary outcomes as odds or risk ratio if reported and associated measures of precision (confidence intervals) or (ii) the raw data needed to calculate odds or risk ratios for primary and secondary outcomes. If reported, adjusted variables reported by authors were also collected for each outcome.

### Synthesis strategy

Study details were summarized descriptively. For the primary outcome, we compared short versus longer-duration antibiotic therapy groups using inverse variance random effects models and the Hartung-Knapp (HKSJ) adjustment method to calculate 95% confidence intervals (CI) [26]. Pooled effect sizes were reported as risk ratios (RR) with 95% CIs and presented as forest plots. We used the Restricted maximum-likelihood (REML) estimator approach to examine between-study variance (tau²). Finally, we also assessed heterogeneity visually in the generated forest plots and using the I [2] statistic (which reflects between-study heterogeneity). While we recognize that cats are not small dogs we opted to do a pooled meta-analysis across species as our primary analysis and examine species effects in sensitivity analyses, since the duration of antibiotics recommended is the same in both species [15]. All analyses were conducted in R statistical software [27].

### Additional analyses

To examine the robustness of findings in our meta-analysis, we completed post-hoc sub-group analyses by species (since cats may be less affected by UTIs compared to dogs [28]), sex (since female dogs and cats are more predisposed to UTIs [29]) and antibiotic duration (since one study compared single-dose therapy to three days of treatment [30], which may also be considered short duration). For the sex sub-group analysis, raw data for female and male animals was used. Within study differences could not be pooled since one study was comprised of exclusively female animals. For all subgroup analyses, we used inverse variance random effects models and the Wald method to calculate 95% CIs [31]. The Wald method was used to calculate CIs for sub-group analyses since the HKSJ method adds additional between-subgroup heterogeneity. In these analyses, sub-groups were very small (1 or 2 studies). Pooled effect sizes were reported as RR with 95% CIs and presented visually as forest plots. For all subgroup analyses, we also assessed heterogeneity visually in the generated forest plots, using the I [2] statistic and examined between-study variance using tau². We planned to apply the Instrument for Assessing the Credibility of Effect Modification Analyses (ICEMAN) tool to assess the credibility of any subgroup analyses if *p* < 0.10.

Sensitivity analyses were performed to evaluate assumptions made when designing this systematic review. Sensitivity analyses were completed in the same manner as the primary meta-analysis. We evaluated the impact of study design and species on meta-analysis results by removing any observational and cat studies to examine randomized control trials and dog-only studies separately.

### Certainty of evidence and publication bias

The quality of evidence for the primary outcome was assessed using the Grading of Recommendations Assessment, Development and Evaluation (GRADE) system [32], and findings were presented as a summary table. GRADEpro software was used to calculate anticipated absolute effects of our risk ratio on 1000 animals [33]. Publication bias was assessed visually with a funnel plot.

### Deviations from protocol

For our primary meta-analysis, the Hartung-Knapp method was chosen (in deviation from our registered protocol) since it can help correct for small-sample bias in the random effects model [26]. A second deviation from our protocol is that we used the Restricted maximum-likelihood (REML) estimator since it also performs better with small sample sizes to examine between-study variance (tau²) [34]. Finally, although we planned to exclude published conference abstracts, due to the limited number of studies identified, we opted to include abstracts which met other study selection criteria.

## Results

### Study selection

**Fig. 1:**
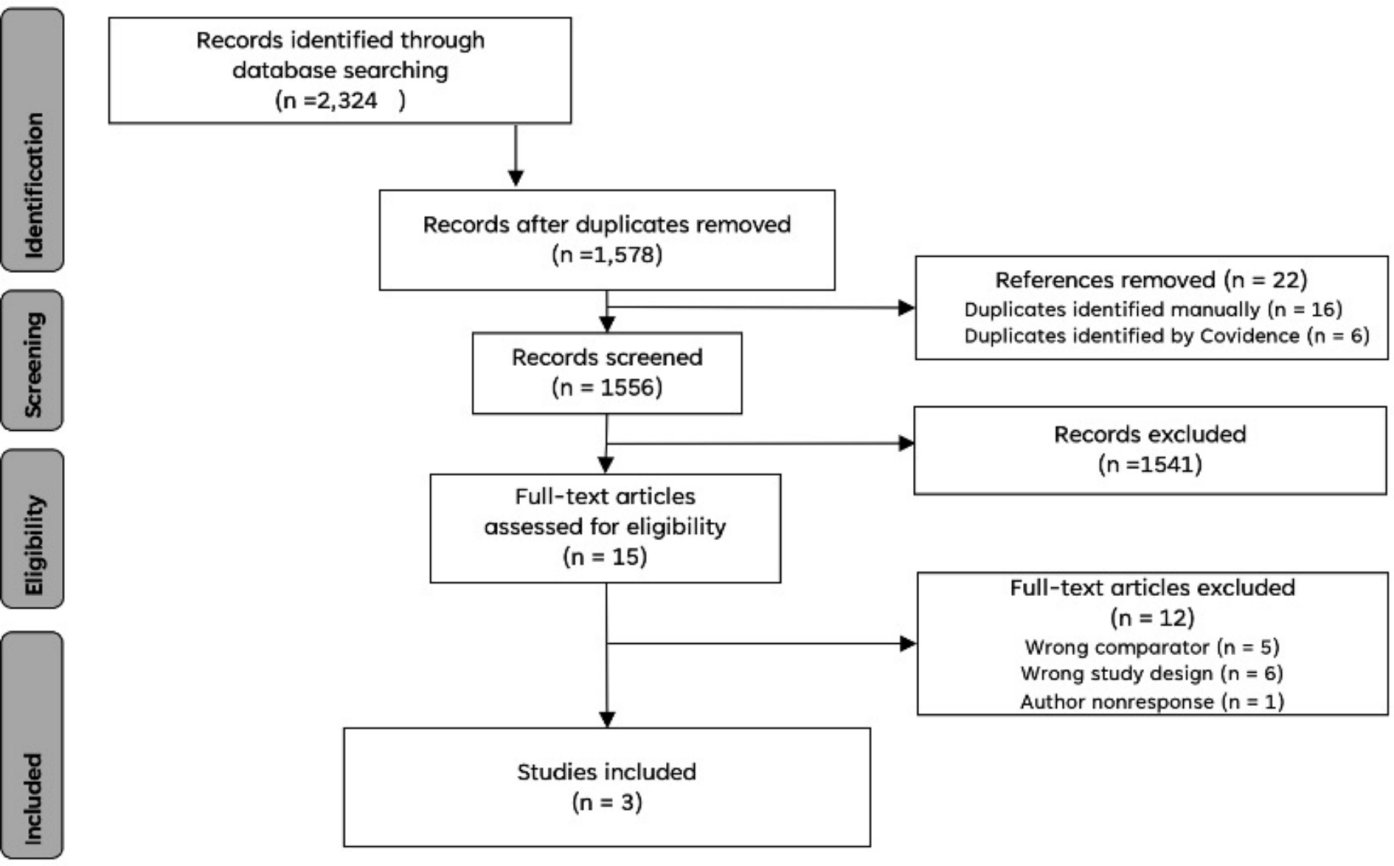
A PRISMA flow diagram depicting the study selection process and exclusion reasons

Of 2,324 citations screened, we identified four studies which met inclusion criteria [30, 35–37], however, one of these was excluded due to missing data and nonresponse from authors [37]. No additional studies were identified from hand-searching the reference lists of included studies. This study reported results combined across different durations and antibiotic types and could not be included in any analyses [37]. A PRISMA [38] flow chart (Fig. 1) depicts the study selection process and exclusion reasons.

### Study characteristics and findings

The three included studies evaluated 54 animals. Two studies looked at UTIs in dogs [30, 35]and one study at UTIs in cats [36]. All were conducted in laboratory animals and looked at the impact of different antibiotic durations on experimentally induced UTIs. Studies varied by antibiotic type, dose and duration. Two studies looked at durations of trimethoprim sulfadiazine [30, 35] one at amikacin [30] and one at amoxicillin [36] durations. No identified studies were published after 1990. Two studies examined single-dose therapy [30, 35], and one looked at three days as their shorter course [36]. Finally, since one study examined short versus long duration of therapy for two different antibiotics [30], each antibiotic was separately analyzed and each antibiotic is reported separately in Table 2. All included studies examined microbiological cure rates as their outcome. Full details on the characteristics of each study can be found in Table 2.

The first study examined the impact of single versus three days of trimethoprim sulfadiazine and amikacin on induced UTIs in mixed-breed dogs, finding microbiological cures were higher on day three, especially for dogs treated with trimethoprim sulfadiazine [30]. The second study examined three days versus 14 days of amoxicillin on induced UTIs in cats, finding slightly higher microbiological cures on day 14^36^. Finally, the third study examined microbiological cure rates of single-dose versus 21 days of trimethoprim sulfadiazine therapy on induced UTIs in only female dogs finding higher cure rates in animals treated with longer durations [35].

**Table 2:** Characteristics of studies included in this review

Last name first author, publication year		Rogers, 1988 (Amikacin)	Rogers, 1988 (Trimethoprim Sulfamethoxazole)	Mann, 1990	Turnwald, 1986
**General Information**	**Study design**	Randomized controlled trial	Randomized controlled trial	Abstract, Experimental trial (randomization not fully described)	Randomized controlled trial
	**Country and setting**	United States, laboratory animals	United States, laboratory animals	United States, laboratory animals	United States, laboratory animals
	**Antibiotic comparison short arm**	Single dose of amikacin treatment	Single dose of trimethoprim sulfadiazine treatment	Three-days of amoxicillin therapy	Single dose of trimethoprim sulfadiazine therapy
	**Antibiotic comparison long arm**	Three days of amikacin treatment	Three days of trimethoprim sulfadiazine treatment	Fourteen days of amoxicillin therapy	Twenty-one days of trimethoprim sulfadiazine therapy
**Population and disease details**	**Species**	Dogs	Dogs	Cats	Dogs
	**Total number of animals included in both short and long arms (sex)**	12 mixed breed dogs (6 males and 6 females)	12 mixed breed dogs (6 males and 6 females)	12 cats (6 males, 6 females*)	18 mixed-breed female dogs
	**Health status of animals**	Health status was assessed by physical exam, complete blood count, biochemistry fecal float and complete urinalysis	Health status was assessed by physical exam, complete blood count, biochemistry fecal float and complete urinalysis	Described as healthy	Described as healthy - a physical examination, urinalysis, and quantitative urine culture were done on each dog
	**Urinary tract infection details (experimentally induced vs. sporadic)**	Induced	Induced	Induced	Induced
**Primary outcomes and measures**	**Outcome measure (clinical or microbiological cure)**	Microbiological cure	Microbiological cure	Microbiological cure	Microbiological cure
	**Urine collection method**	Urethral catheterization	Urethral catheterization	Not specified	Cystocentesis
**Secondary outcomes**	**Long term cure rate(s)**,** adverse events**	None reported	None reported	None reported	None reported
**Risk of Bias**	**Overall risk of bias**	High	High	Serious	High

*Reported sex data was verified from thesis material published separately from this abstract [39].

### Risk of bias

One study was a trial for which randomization was not fully described [36]. This study was assessed as having serious risk of bias, while the other two were randomized controlled trials, both assessed as having a high risk of bias [30, 35]. A concern in all studies was the use of experimentally induced urinary tract infections as a model for naturally occurring UTIs. The overall risk of bias for each study is listed in Table 2. The risk of bias across each domain is available for each study in *Appendix 4*.

### Meta-analyses

**Fig. 2:**
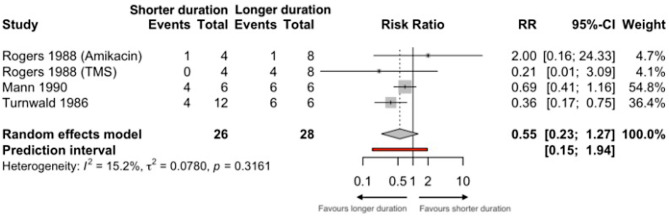
Primary meta-analysis of cure rates with short versus long duration therapy for UTIs in dogs and cats. Since some studies have 0 events, 0.5 was added to all frequency counts in the meta-analysis. TMS is Trimethoprim/sulfamethoxazole

The three studies reported the primary outcome (short versus long-duration antibiotic therapy cure rates for UTIs in dogs and cats) across 54 animals. The pooled risk ratio of short versus long duration of antibiotic therapy indicated lower cure rates with shorter duration treatment, but this was not statistically significant (RR 0.55, 95% CI: 0.23–1.27; very low certainty) (Fig. 2*).* The value of I [2] suggests low heterogeneity (15.2%, 95% CI 0.00–87.0), while tau² (0.078, 95% CI 0.00-11.5) suggests some degree of variability across studies. The large confidence interval of both measures, however, reflects the considerable uncertainty in both estimates. Secondary outcomes of interest, including long-term UTI cure rates or adverse events, were not reported.

**Fig. 3:**
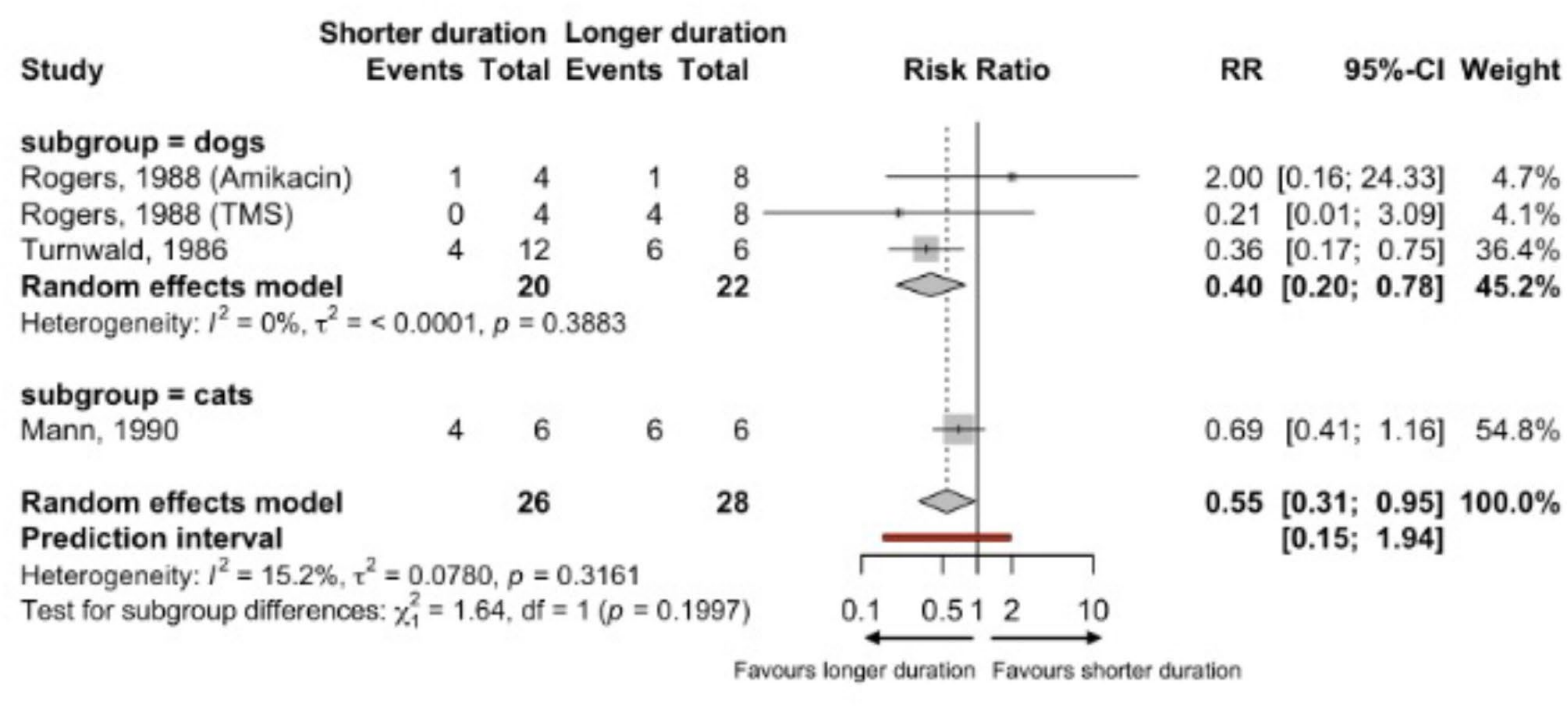
Sub-group meta-analysis results of short versus long duration therapy microbiological cure rates for UTIs in dogs and cats by species. Since some studies have 0 events, 0.5 was added to all frequency counts in the meta-analysis. TMS is Trimethoprim/sulfamethoxazole.

Subgroup analyses identified no significant subgroup effects due to species (*p* = 0.19) (Fig. 3), sex (*p* = 0.14), or duration of therapy (*p* = 0.35) (*Appendix 5*). Since no significant difference in the effect between subgroups was found, we did not apply the ICEMAN tool to assess the credibility of any subgroup analyses. While other animal [40, 41] and disease factors [29] may also affect the robustness of our findings, both dog studies examined induced UTIs in mixed-breed, middle-aged dogs, thus breed and age sub-group analyses were not pursued.

### Sensitivity analysis

Sensitivity analyses by study type and species (dog and randomized controlled studies only) showed similar results to the primary meta-analysis (*Appendix 6*).

### Publication bias

Due to a limited number of identified studies, publication bias was not assessed using the Egger test which will lack power with fewer than 10 studies. A Funnel plot of included studies (with the Rogers et al. study separated by antibiotic type) is available in *Appendix 7*. Visual assessment of the funnel plot does not show obvious asymmetry however due to the lack of studies these findings are less reliable.

### Summary of findings and grade assessment of certainty of evidence

Evidence supporting our primary outcome was evaluated as having a very low certainty of evidence due to a high risk of bias across studies and high indirectness due to most studies using experimentally induced urinary tract infections, which may not be reflective of sporadic simple UTIs and microbiological cure as their outcome instead of clinical cure which is less relevant from a patient, owner and veterinarian perspective. Studies also had high imprecision due to our potential risk of publication bias with only older studies identified and because our confidence intervals reflect that longer durations could be better or worse than shorter durations, making our result uncertain. This imprecision is further reflected in our large anticipated absolute effect range which showed that shorter durations of antibiotics may cure 467 fewer animals or up to 164 more animals than longer durations. While 467 fewer animals cured out of 1000 would be clinically relevant, 164 more might not be. A full summary of the assessment of the certainty of evidence for the primary outcome is available in Table 3.

**Table 3:** Grade assessment of the certainty of evidence for primary outcome

Outcome (importance)	Number of studies (Number of animals)	Certainty assessment						Effect			Certainty	Anticipated absolute effects	
		Study design	Risk of bias	Inconsis-tency	Indirect-ness	Imprecisi-on	Other considerations	Individuals cured on short duration	Individuals cured on long duration	Risk ratio (95% CI)		Risk with long duration	Risk difference with shorter duration
Clinical or microbiological cure of UTIs (important)	3 (54)	2 RCTs and 1 non-randomized clinical trial	serious	not serious	serious	very serious	Not able to assess publication bias Laboratory animals only Experimentally induced UTIs	9 of 26 animals (34.6%)	17 of 28 animals (60.7%)	RR 0.55, (95% CI: 0.23–1.27)	Very low	607 per 1000	273 fewer per 1000 (from 467 fewer to 164 more)

## Discussion

Using current data, we were not able to show significant differences in microbiological cure rates between shorter durations of antibiotics compared to longer durations when treating urinary tract infections in dogs and cats. The meta-analysis of our primary outcome was non-statistically significant (RR 0.55, 95% CI: 0.23–1.27; very low certainty). A small sample size of animals included in studies (with only a total of 54 animals) may have contributed to our findings. Additionally, that studies examined different durations and only considered microbiological cures may have also influenced these findings. We were not able to examine any secondary outcomes, such as long-term UTI cure rates or adverse events, as these outcomes were not reported in the included studies. For adverse events, this may reflect that either the studies did not capture this outcome or that none occurred.

No other meta-analyses have examined the efficacy of different antibiotic therapy durations for treating urinary tract infections in dogs and cats. The lack of studies found by this review echoes the lack of evidence highlighted by ISCAID guidelines on treating UTIs in dogs and cats [15] and the findings of a similar 2015 systematic review in dogs [18], which was unable to perform a meta-analysis since not enough studies were identified.

### Limitations

Firstly, while measures of between-study heterogeneity and variance were low, confidence intervals for these measures were large, meaning both measures are uncertain. Included studies varied by species (cats and dogs), study design and antibiotic type, dose and duration. For the duration, all three studies compared different durations, with one study comparing single-dose therapy to three doses, which could still be considered short-duration. Additionally, the other studies both examined quite long durations in their long arms with one study comparing single-dose therapy to 21 days of therapy, a much longer duration than would traditionally be used for sporadic bacterial UTIs. In women, single-dose therapy has been shown to be less effective in treating uncomplicated cystitis, although this may depend on the antibiotic and dose [42, 43].

Secondly, the studies included in this review may not have accurately captured the population and disease of interest. Studies included male and female animals, even though UTIs are more common in female animals [44]. Additionally, urinary tract infections in male animals are more likely to involve the prostate and require longer durations of antibiotics [15]. All included studies also used experimentally induced UTIs. Induced UTIs are likely not reflective of naturally occurring sporadic bacterial UTIs, and reported cure rates may not reflect cure rates for naturally occurring UTIs. Two of the studies induced bacterial infections using *Staphylococcus intermedius*, whereas *Escherichia coli* is the most common bacteria isolated from urinary tract infections in pets [45]. Finally, all three studies looked at microbiological cures as their outcome; future trials examining clinical cures as reported by owners will be more pragmatic and relevant to both veterinarians, owners and the animals themselves For owners and veterinarians clinical cure is a more relevant outcome when each urinalysis and/or culture represents an additional vet visit and cost. For the patients or animals clinical cure is also a more useful outcome since an unsuccessful microbiological cure in the absence of clinical signs is classified as subclinical bacteriuria and should not require further antibiotic treatment [15].

Like most systematic reviews, publication bias may also have impacted our results. Although we designed our search strategy to search across multiple sources, included studies published at any time and hand-searched across reference lists of identified studies, few and only older studies were identified. This smaller number of studies included means our funnel plot, which was symmetrical, should be interpreted cautiously and may be inaccurate.

While we were not able to show significant differences in microbiological cure rates between shorter and longer durations of antibiotic therapy these findings do not in any way contradict the updated ISCAID guidelines which recommend 3 to 5 days of antibiotic therapy for treating sporadic UTIs in dogs and cats [15]. In humans, similar durations are well supported [46, 47]. In order to make clear clinical recommendations to veterinarians going forward, our findings highlight the need for additional, high-quality studies from veterinary settings.

## Conclusions

High-quality evidence will inform clinical guidelines and modernize the clinical practice of small animal veterinarians. The prudent use of antimicrobials is essential for maintaining the effectiveness of antimicrobials for use in both humans and animals [48], which is why the World Health Organization has prioritized addressing antimicrobial use in animals [49]. Our findings highlight the need for additional high-quality, larger clinical trials examining antibiotic duration for treating simple, naturally occurring UTIs in both dogs and cats. Secondary outcomes such as long-term UTI cure rates should also be investigated, and adverse events should be explicitly reported. These studies will support veterinary clinical decision-making, inform clinical guidelines and ultimately improve antimicrobial stewardship in veterinary medicine.

## Electronic supplementary material

Below is the link to the electronic supplementary material.

Supplementary Material 1

## Data Availability

All data generated or analyzed during this study are included in this published article and its supplementary information files.
