# Supplementary material for: Short versus longer duration antibiotic treatment for urinary tract infections in companion animals: a systematic review and meta-analysis
Source: BMC Vet Res. 2025 Apr 17;21:280. doi: 10.1186/s12917-025-04722-y (PMC12004815; doi:10.1186/s12917-025-04722-y)
Supplement: Supplementary file 1 — Supplementary Material 1 [file 12917_2025_4722_MOESM5_ESM.docx]

**Appendix 1 – PRISMA-P Checklist**

| **Section and Topic** | **Item #** | **Checklist item** | **Location where item is reported** |
| --- | --- | --- | --- |
| **TITLE** | | |  |
| Title | 1 | Identify the report as a systematic review. | Pg.1 |
| **ABSTRACT** | | |  |
| Abstract | 2 | See the PRISMA 2020 for Abstracts checklist. | Pg.1-3 |
| **INTRODUCTION** | | |  |
| Rationale | 3 | Describe the rationale for the review in the context of existing knowledge. | Pg. 4 |
| Objectives | 4 | Provide an explicit statement of the objective(s) or question(s) the review addresses. | Pg. 5 |
| **METHODS** | | |  |
| Eligibility criteria | 5 | Specify the inclusion and exclusion criteria for the review and how studies were grouped for the syntheses. | Pg. 5-6 |
| Information sources | 6 | Specify all databases, registers, websites, organisations, reference lists and other sources searched or consulted to identify studies. Specify the date when each source was last searched or consulted. | Pg. 6-7 |
| Search strategy | 7 | Present the full search strategies for all databases, registers and websites, including any filters and limits used. | Pg. 7, Appendix 2 |
| Selection process | 8 | Specify the methods used to decide whether a study met the inclusion criteria of the review, including how many reviewers screened each record and each report retrieved, whether they worked independently, and if applicable, details of automation tools used in the process. | Pg. 7 |
| Data collection process | 9 | Specify the methods used to collect data from reports, including how many reviewers collected data from each report, whether they worked independently, any processes for obtaining or confirming data from study investigators, and if applicable, details of automation tools used in the process. | Pg. 7-8 |
| Data items | 10a | List and define all outcomes for which data were sought. Specify whether all results that were compatible with each outcome domain in each study were sought (e.g. for all measures, time points, analyses), and if not, the methods used to decide which results to collect. | Pg. 8, Appendix 3 |
|  | 10b | List and define all other variables for which data were sought (e.g. participant and intervention characteristics, funding sources). Describe any assumptions made about any missing or unclear information. | Pg. 8, Appendix 3 |
| Study risk of bias assessment | 11 | Specify the methods used to assess risk of bias in the included studies, including details of the tool(s) used, how many reviewers assessed each study and whether they worked independently, and if applicable, details of automation tools used in the process. | Pg. 8 |
| Effect measures | 12 | Specify for each outcome the effect measure(s) (e.g. risk ratio, mean difference) used in the synthesis or presentation of results. | Pg. 9 |
| Synthesis methods | 13a | Describe the processes used to decide which studies were eligible for each synthesis (e.g. tabulating the study intervention characteristics and comparing against the planned groups for each synthesis (item #5)). | Pg. 9 |
|  | 13b | Describe any methods required to prepare the data for presentation or synthesis, such as handling of missing summary statistics, or data conversions. | Pg. 9-10 |
|  | 13c | Describe any methods used to tabulate or visually display results of individual studies and syntheses. | Pg. 9-10 |
|  | 13d | Describe any methods used to synthesize results and provide a rationale for the choice(s). If meta-analysis was performed, describe the model(s), method(s) to identify the presence and extent of statistical heterogeneity, and software package(s) used. | Pg. 9-10 |
|  | 13e | Describe any methods used to explore possible causes of heterogeneity among study results (e.g. subgroup analysis, meta-regression). | Pg. 10 |
|  | 13f | Describe any sensitivity analyses conducted to assess robustness of the synthesized results. | Pg. 10-11 |
| Reporting bias assessment | 14 | Describe any methods used to assess risk of bias due to missing results in a synthesis (arising from reporting biases). | Pg. 10 |
| Certainty assessment | 15 | Describe any methods used to assess certainty (or confidence) in the body of evidence for an outcome. | Pg. 11 |
| **RESULTS** | | |  |
| Study selection | 16a | Describe the results of the search and selection process, from the number of records identified in the search to the number of studies included in the review, ideally using a flow diagram. | Pg.11 |
|  | 16b | Cite studies that might appear to meet the inclusion criteria, but which were excluded, and explain why they were excluded. | Pg. 12 |
| Study characteristics | 17 | Cite each included study and present its characteristics. | Pg. 13 |
| Risk of bias in studies | 18 | Present assessments of risk of bias for each included study. | Pg. 14, Appendix 4 |
| Results of individual studies | 19 | For all outcomes, present, for each study: (a) summary statistics for each group (where appropriate) and (b) an effect estimate and its precision (e.g. confidence/credible interval), ideally using structured tables or plots. | Pg. 13-14 |
| Results of syntheses | 20a | For each synthesis, briefly summarise the characteristics and risk of bias among contributing studies. | Pg. 14 |
|  | 20b | Present results of all statistical syntheses conducted. If meta-analysis was done, present for each the summary estimate and its precision (e.g. confidence/credible interval) and measures of statistical heterogeneity. If comparing groups, describe the direction of the effect. | Pg. 14  Appendix 5 |
|  | 20c | Present results of all investigations of possible causes of heterogeneity among study results. | Pg. 14 |
|  | 20d | Present results of all sensitivity analyses conducted to assess the robustness of the synthesized results. | Pg. 15  Appendix 6 |
| Reporting biases | 21 | Present assessments of risk of bias due to missing results (arising from reporting biases) for each synthesis assessed. | Pg. 15, Appendix 7 |
| Certainty of evidence | 22 | Present assessments of certainty (or confidence) in the body of evidence for each outcome assessed. | Pg. 15-16 |
| **DISCUSSION** | | |  |
| Discussion | 23a | Provide a general interpretation of the results in the context of other evidence. | Pg. 16-17 |
|  | 23b | Discuss any limitations of the evidence included in the review. | Pg. 17-18 |
|  | 23c | Discuss any limitations of the review processes used. | Pg. 18 |
|  | 23d | Discuss implications of the results for practice, policy, and future research. | Pg. 18 |
| **OTHER INFORMATION** | | |  |
| Registration and protocol | 24a | Provide registration information for the review, including register name and registration number, or state that the review was not registered. | Pg. 5 |
|  | 24b | Indicate where the review protocol can be accessed, or state that a protocol was not prepared. | Pg. 5 |
|  | 24c | Describe and explain any amendments to information provided at registration or in the protocol. | Pg. 11 |
| Support | 25 | Describe sources of financial or non-financial support for the review, and the role of the funders or sponsors in the review. | Pg. 19 |
| Competing interests | 26 | Declare any competing interests of review authors. | Pg. 19 |
| Availability of data, code and other materials | 27 | Report which of the following are publicly available and where they can be found: template data collection forms; data extracted from included studies; data used for all analyses; analytic code; any other materials used in the review. | Pg. 20 |

*From:*  Page MJ, McKenzie JE, Bossuyt PM, Boutron I, Hoffmann TC, Mulrow CD, et al. The PRISMA 2020 statement: an updated guideline for reporting systematic reviews. BMJ 2021;372:n71. doi: 10.1136/bmj.n71. This work is licensed under CC BY 4.0. To view a copy of this license, visit <https://creativecommons.org/licenses/by/4.0/>

**Appendix 2 –Search Strategies**

MEDLINE

Ovid MEDLINE(R) ALL <1946 to October 01, 2024>

| # | Searches |
| --- | --- |
| 1 | Antibiotic Stewardship/ or Anti-Bacterial Agents/ or Anti-Infective Agents/ or Amikacin/ or Amoxicillin/ or Clavulanic Acid/ or Ampicillin/ or Caphalosporins/ or Cephalexin/ or Chloramphenicol/ or Doxycycline/ or Enrofloxacin/ or Fluoroquinolones/ or Nitrofurantoin/ or Sulfisoxazole/ or Tetracycline/ or Trimethoprim, Sulfamethoxazole Drug Combination/ or (anti-infective* or antiinfective* or anti-biotic* or antibiotic* or anti-microbial* or antimicrobial* or antibacterial* or anti-bacterial* or amikacin or amoxicillin or clavulanate or ampicillin or cefovecin or cephalexin or chloramphenicol or doxycycline or enrofloxacin or marbofloxacin or meropenem or nitrofurantoin or sulfisoxazole or tetracycline or "trimethoprim/sulfadiazine" or fluoroquinolone* or caphalosporin*).ti,kw,kf. |
| 2 | Urinary Tract Infections/ or Bacteriuria/ or Pyuria/ or Pyelonephritis/ or Prostatitis/ or Cystitis/ or Cystitis, Interstitial/ or Pyelocystitis/ or ((("urinary tract" or urologic* or bladder) adj5 (infection* or pathogen* or bacteria)) or bacteriuria or pyuria or cystitis or prostatitis or pyelonephritis).ab,ti,kw,kf. or (UTI or UTIs).ti. |
| 3 | Bacteriuria/dt or Cystitis/dt or Cystitis, Interstitial/dt or Prostatitis/dt or Pyelocystitis/dt or Pyelonephritis/dt or Pyuria/dt or Urinary Tract Infections/dt |
| 4 | 1 and 2 |
| 5 | 3 or 4 |
| 6 | Cat Diseases/ or Cats/ or Dog Diseases/ or Dogs/ or ("small animal*" or "companion animal*" or pet or pets or dog or dogs or canine* or bitch* or puppy or pup* or puppies or hound* or mongrel* or cat or cats or feline* or tom or tomcat* or kitten* or queen or ((pet? or veterinar*) adj (clinic or clinics or hospital or hospitals))).ab,ti,kw,kf. |
| 7 | 5 and 6 |
| 8 | Bacteriuria/ve or Cystitis/ve or Cystitis, Interstitial/ve or Prostatitis/ve or Pyelocystitis/ve or Pyelonephritis/ve or Pyuria/ve or Urinary Tract Infections/ve |
| 9 | 1 and 8 |
| 10 | 7 or 9 |
| 11 | Time Factors/ or Drug Administration Schedule/ or (dose? or dosing or dosage or duration or therapy or therapies or treatment* or therapeutic* or regimen* or prescrib* or prescription* or management or overprescrib* or duration or time or length or "day" or days or week* or short-course or shorter-course or short-term or shorter-term or short-duration or long-course or longer-course or long-term or longer-term or long-duration or second-course or overuse or overprescrib* or therapy or therapies or ((minimum or maximum or standard* or average or prolong* or extend* or increas* or recommend* or suggest* or guideline* or guidance or optimal or optimiz* or reduce* or reduction or decreas* or fixed or defined or full or complet* or finish* or continue* or repeat* or discontinue* or number or "7" or seven) adj3 (course or term* or duration or time or length or day* or week* or schedule* or regimen*))).ab,ti,kf,kw. |
| 12 | 10 and 11 |
| 13 | remove duplicates from 12 |

Embase

Ovid Embase <1974 to 2024 October 01>

| # | Searches |
| --- | --- |
| 1 | exp antibiotic agent/ or antiinfective agent/ or antimicrobial stewardship/ or (anti-infective* or antiinfective* or anti-biotic* or antibiotic* or anti-microbial* or antimicrobial* or antibacterial* or anti-bacterial* or amikacin or amoxicillin or clavulanate or ampicillin or cefovecin or cephalexin or chloramphenicol or doxycycline or enrofloxacin or marbofloxacin or meropenem or nitrofurantoin or sulfisoxazole or tetracycline or "trimethoprim/sulfadiazine" or fluoroquinolone* or caphalosporin*).ti,kw,kf. |
| 2 | bacteriuria/ or cystitis/ or kidney infection/ or pyonephrosis/ or prostatisis/ or urinary tract infection/ or urinary tract inflammation/ or ((("urinary tract" or urologic* or bladder) adj5 (infection* or pathogen* or bacteria)) or bacteriuria or pyuria or cystitis or prostatitis or pyelonephritis).ab,ti. or (UTI or UTIs).ti. |
| 3 | bacteriuria/dt or cystitis/dt or kidney infection/dt or pyonephrosis/dt or prostatisis/dt or urinary tract infection/dt or urinary tract inflammation/dt |
| 4 | (1 and 2) or 3 |
| 5 | cat disease/ or cat/ or exp cat breed/ or domestic cat/ or feral cat/ or kitten/ or "queen (cat)"/ or stray cat/ or tomcat/ or dog disease/ or dog/ or exp dog breed/ or feral dog/ or puppy/ or stray dog/ or exp working dog/ or ("small animal*" or "companion animal*" or dog or dogs or canine* or bitch* or puppy or pup or pups or puppies or hound* or mongrel* or cat or cats or feline* or tom or tomcat* or kitten* or queen or ((pet? or veterinar*) adj (clinic or clinics or hospital or hospitals))).ab,ti,kw,kf. |
| 6 | drug administration/ or time factor/ or (dose? or dosing or dosage or duration or therapy or therapies or treatment* or therapeutic* or regimen* or prescrib* or prescription* or management or overprescrib* or duration or time or length or "day" or days or week* or short-course or shorter-course or short-term or shorter-term or short-duration or long-course or longer-course or long-term or longer-term or long-duration or second-course or overuse or overprescrib* or therapy or therapies or ((minimum or maximum or standard* or average or prolong* or extend* or increas* or recommend* or suggest* or guideline* or guidance or optimal or optimiz* or reduce* or reduction or decreas* or fixed or defined or full or complet* or finish* or continue* or repeat* or discontinue* or number or "7" or seven) adj3 (course or term* or duration or time or length or day* or week* or schedule* or regimen*))).ab,ti,kf,kw. |
| 7 | 4 and 5 and 6 |
| 8 | remove duplicates from 7 |

CAB

Ovid CAB Abstracts <1973 to 2024 Week 39>

| # | Searches |
| --- | --- |
| 1 | exp antibiotics/ or antibacterial agents/ or antiinfective agents/ or (anti-infective* or antiinfective* or anti-biotic* or antibiotic* or anti-microbial* or antimicrobial* or antibacterial* or anti-bacterial* or amikacin or amoxicillin or clavulanate or ampicillin or cefovecin or cephalexin or chloramphenicol or doxycycline or enrofloxacin or marbofloxacin or meropenem or nitrofurantoin or sulfisoxazole or tetracycline or "trimethoprim/sulfadiazine" or fluoroquinolone* or caphalosporin*).ti,ab,id. |
| 2 | bacteriuria/ or cystitis/ or prostatitis/ or pyelonephritis/ or urethritis/ or urinary tract infections/ or ((("urinary tract" or urologic* or bladder) adj5 (infection* or pathogen* or bacteria)) or bacteriuria or pyuria or cystitis or prostatitis or pyelonephritis).mp. or (UTI or UTIs).ti. |
| 3 | LL070.cc. or canis/ or cats/ or cat diseases/ or kittens/ or feral cats/ or exp dogs/ or dog diseases/ or felis/ or pets/ or ("small animal*" or "companion animal*" or pet or pets or dog or dogs or canine* or bitch* or puppy or pup* or puppies or hound* or mongrel* or cat or cats or feline* or tom or tomcat* or kitten* or queen or ((pet? or veterinar*) adj (clinic or clinics or hospital or hospitals))).ti,ab,id. |
| 4 | dosage/ or drug therapy/ or overdose/ or time/ or (dose? or dosing or dosage or duration or therapy or therapies or treatment* or therapeutic* or regimen* or prescrib* or prescription* or management or overprescrib* or duration or time or length or "day" or days or week* or short-course or shorter-course or short-term or shorter-term or short-duration or long-course or longer-course or long-term or longer-term or long-duration or second-course or overuse or overprescrib* or therapy or therapies or ((minimum or maximum or standard* or average or prolong* or extend* or increas* or recommend* or suggest* or guideline* or guidance or optimal or optimiz* or reduce* or reduction or decreas* or fixed or defined or full or complet* or finish* or continue* or repeat* or discontinue* or number or "7" or seven) adj3 (course or term* or duration or time or length or day* or week* or schedule* or regimen*))).ti,ab,id. |
| 5 | 1 and 2 and 3 and 4 |
| 6 | remove duplicates from 5 |

Scopus

Name of database (long)

| # | Search |
| --- | --- |
| 1 | TITLE-ABS-KEY ( anti-infective* OR antiinfective* OR anti-biotic* OR antibiotic* OR anti-microbial* OR antimicrobial* OR antibacterial* OR anti-bacterial* OR amikacin OR amoxicillin OR clavulanate OR ampicillin OR cefovecin OR cephalexin OR chloramphenicol OR doxycycline OR enrofloxacin OR marbofloxacin OR meropenem OR nitrofurantoin OR sulfisoxazole OR tetracycline OR "trimethoprim/sulfadiazine" OR fluoroquinolone* OR caphalosporin ) |
| 2 | TITLE-ABS-KEY ( ( ( "urinary tract" OR urologic* OR bladder ) W/5 ( infection* OR pathogen* OR bacteria ) ) OR bacteriuria OR pyuria OR cystitis OR prostatitis OR pyelonephritis ) |
| 3 | TITLE ( utis OR uti ) |
| 4 | 2 or 3 |
| 5 | TITLE-ABS-KEY ( "small animal*" OR "companion animal*" OR dog OR dogs OR canine* OR bitch* OR puppy OR pup OR pups OR puppies OR hound* OR mongrel* OR cat OR cats OR feline* OR tom OR tomcat* OR kitten* OR queen OR ( ( pet OR pets OR veterinar* ) W/1 ( clinic OR clinics OR hospital OR hospitals ) ) ) |
| 6 | TITLE-ABS-KEY ( dose* OR dosing OR dosage OR duration OR therapy OR therapies OR treatment* OR therapeutic* OR regimen* OR prescrib* OR prescription* OR management OR overprescrib* OR duration OR time OR length OR "day" OR days OR week* OR short-course OR shorter-course OR short-term OR shorter-term OR short-duration OR long-course OR longer-course OR long-term OR longer-term OR long-duration OR second-course OR overuse OR overprescrib* OR therapy OR therapies OR ( ( minimum OR maximum OR standard* OR average OR prolong* OR extend* OR increas* OR recommend* OR suggest* OR guideline* OR guidance OR optimal OR optimiz* OR reduce* OR reduction OR decreas* OR fixed OR defined OR full OR complet* OR finish* OR continue* OR repeat* OR discontinue* OR number OR "7" OR seven ) W/3 ( course OR term* OR time OR length OR day* OR week* OR schedule* OR regimen* ) ) ) |
| 7 | 1 and 4 and 5 and 6 |
| 8 | Embase records removed |
|  | **Search as it appeared in Scopus:** |
|  | ( TITLE-ABS-KEY ( anti-infective* OR antiinfective* OR anti-biotic* OR antibiotic* OR anti-microbial* OR antimicrobial* OR antibacterial* OR anti-bacterial* OR amikacin OR amoxicillin OR clavulanate OR ampicillin OR cefovecin OR cephalexin OR chloramphenicol OR doxycycline OR enrofloxacin OR marbofloxacin OR meropenem OR nitrofurantoin OR sulfisoxazole OR tetracycline OR "trimethoprim/sulfadiazine" OR fluoroquinolone* OR caphalosporin ) ) AND ( TITLE-ABS-KEY ( "small animal*" OR "companion animal*" OR dog OR dogs OR canine* OR bitch* OR puppy OR pup OR pups OR puppies OR hound* OR mongrel* OR cat OR cats OR feline* OR tom OR tomcat* OR kitten* OR queen OR ( ( pet OR pets OR veterinar* ) W/1 ( clinic OR clinics OR hospital OR hospitals ) ) ) ) AND ( TITLE-ABS-KEY ( dose* OR dosing OR dosage OR duration OR therapy OR therapies OR treatment* OR therapeutic* OR regimen* OR prescrib* OR prescription* OR management OR overprescrib* OR duration OR time OR length OR "day" OR days OR week* OR short-course OR shorter-course OR short-term OR shorter-term OR short-duration OR long-course OR longer-course OR long-term OR longer-term OR long-duration OR second-course OR overuse OR overprescrib* OR therapy OR therapies OR ( ( minimum OR maximum OR standard* OR average OR prolong* OR extend* OR increas* OR recommend* OR suggest* OR guideline* OR guidance OR optimal OR optimiz* OR reduce* OR reduction OR decreas* OR fixed OR defined OR full OR complet* OR finish* OR continue* OR repeat* OR discontinue* OR number OR "7" OR seven ) W/3 ( course OR term* OR time OR length OR day* OR week* OR schedule* OR regimen* ) ) ) ) AND ( ( TITLE-ABS-KEY ( ( ( "urinary tract" OR urologic* OR bladder ) W/5 ( infection* OR pathogen* OR bacteria ) ) OR bacteriuria OR pyuria OR cystitis OR prostatitis OR pyelonephritis ) ) OR ( TITLE ( utis OR uti ) ) ) AND NOT INDEX ( embase ) |

**Appendix 3 - Data extraction**

*Table 2. Data items*

| **General Information** | **Last name first author** |
| --- | --- |
|  | Year of Publication |
|  | Last name of primary contact author |
|  | Study design |
|  | Country and setting |
|  | Study aim |
|  | Ethics approval |
|  | Funding Source |
| **Population and disease details** | Species |
|  | Number of animals included |
|  | Health status of animals |
|  | Animal signalment if reported (age/sex) |
|  | Urinary tract infection details (induced vs sporadic) and how this was assessed |
| **Antimicrobial intervention and comparator** | Antibiotic intervention (drug, dose and duration (intent to treat duration and achieved duration if reported)) |
|  | Antibiotic comparator(s) (if control group or multiple therapy durations compared – number of comparison groups, drug(s), dose(s) and duration(s)) |
|  | Outcome measure (clinical or microbiological cure) |
|  | Urine collection method and date(s) of sampling (cystocentesis or catheterization or free catch) |
| **Primary outcomes and measures** | Effect measures (Odds ratio or risk ratio) |
|  | Measures of significance associated with odds ratio or risk ratio (confidence intervals) |
|  | Adjusted variables if reported by authors |
| **OR Data needed to calculate Odds Ratio for primary outcome** | Number of animals on short duration antibiotic therapy |
|  | Number of animals cured (clinical or microbiological) while on short duration antibiotic |
|  | Number of animals on long duration antibiotic therapy |
|  | Number of animals cured (clinical or microbiological) while on long duration antibiotic |
| **Secondary outcomes** | Long term cure rate(s), urine collection method and date(s) of sampling |
|  | Mortality |
|  | Other adverse events, number of animals affected and event severity |
|  | Other secondary outcomes as reported by authors |
| **Additional Comments** | Miscellaneous comments from the study authors or by the review authors |

**Appendix 4 - Risk of Bias**

#
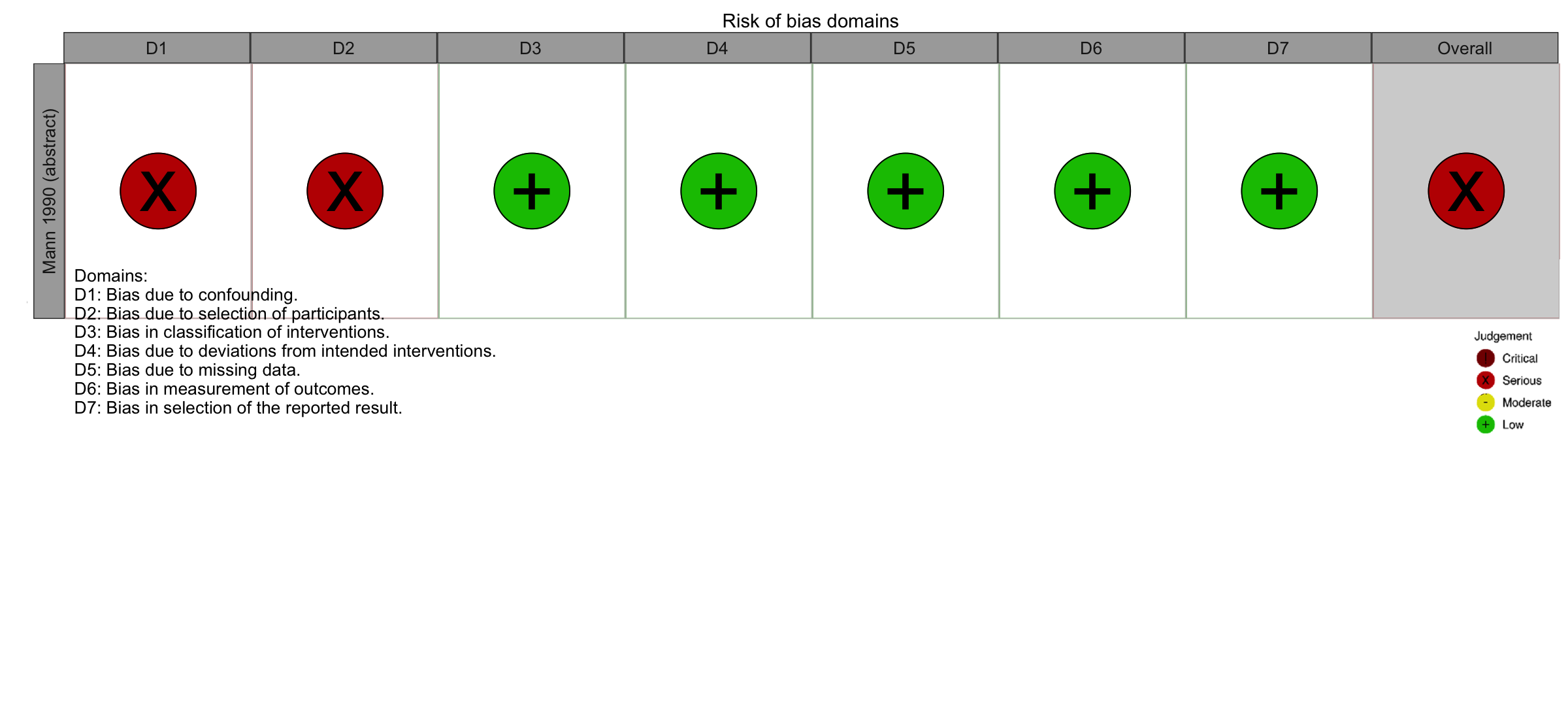


# Figure S1. Traffic-light plot for the risk of bias domains in ROBINS-I for the relevant study

**
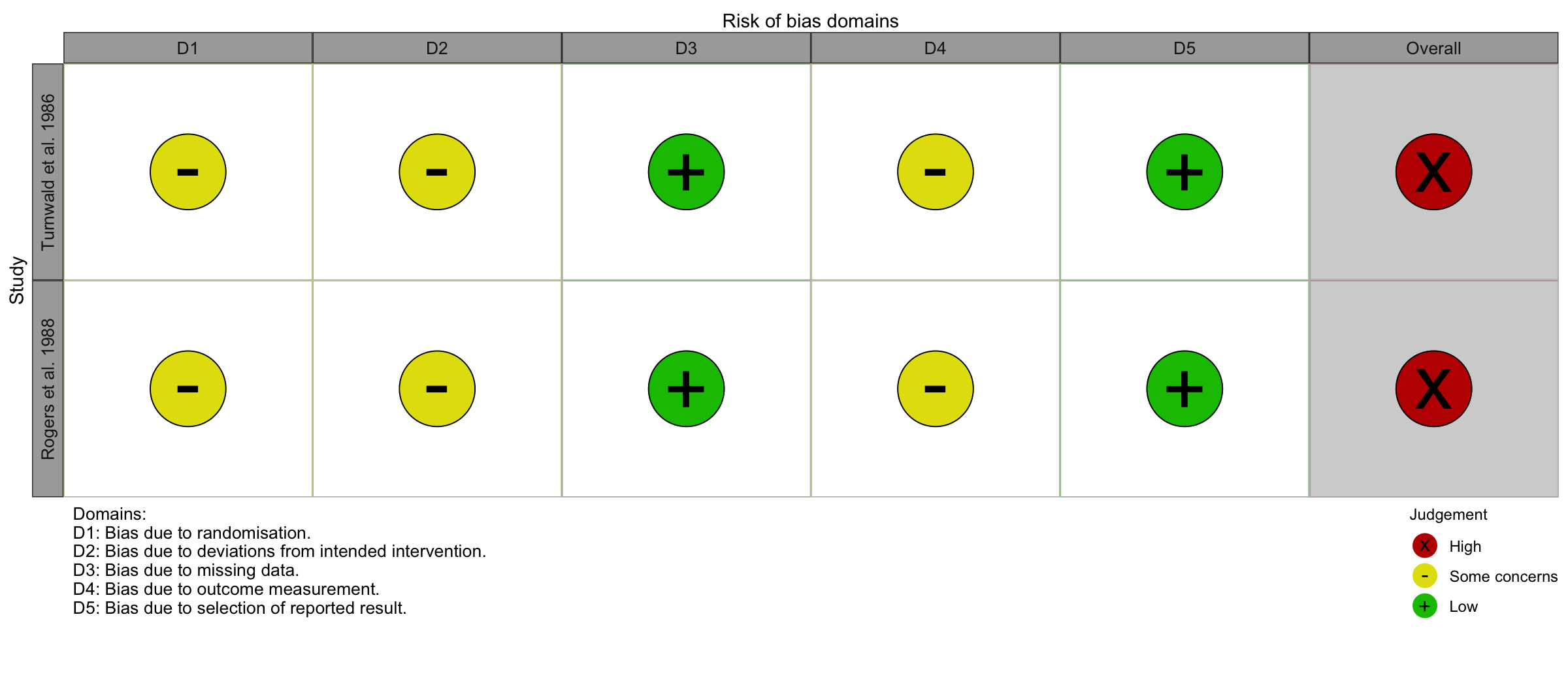
**

# Figure S2. Traffic-light plot for the risk of bias domains in ROB2 for the relevant studies

**Appendix 5 - Sub-group analyses**


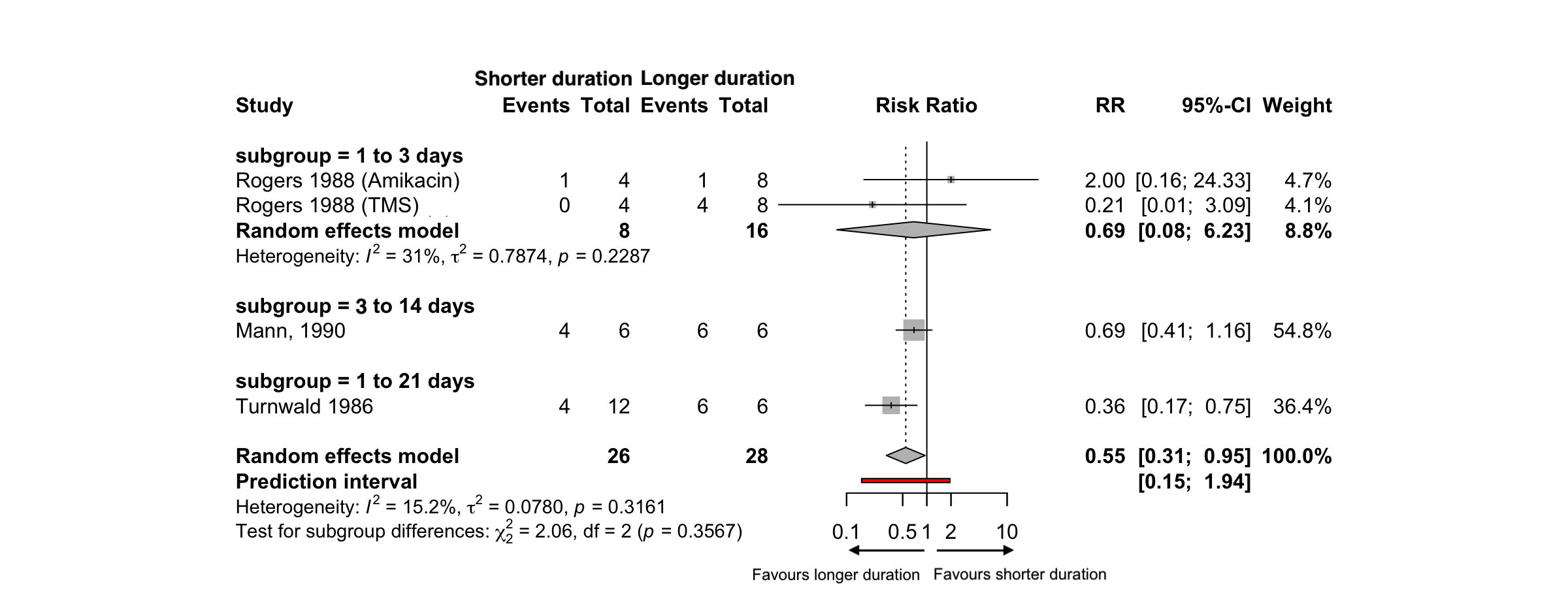
Figure S3. Sub-group meta-analysis results of short versus long duration therapy microbiological cure rates for UTIs in dogs and cats by antibiotic duration group

**
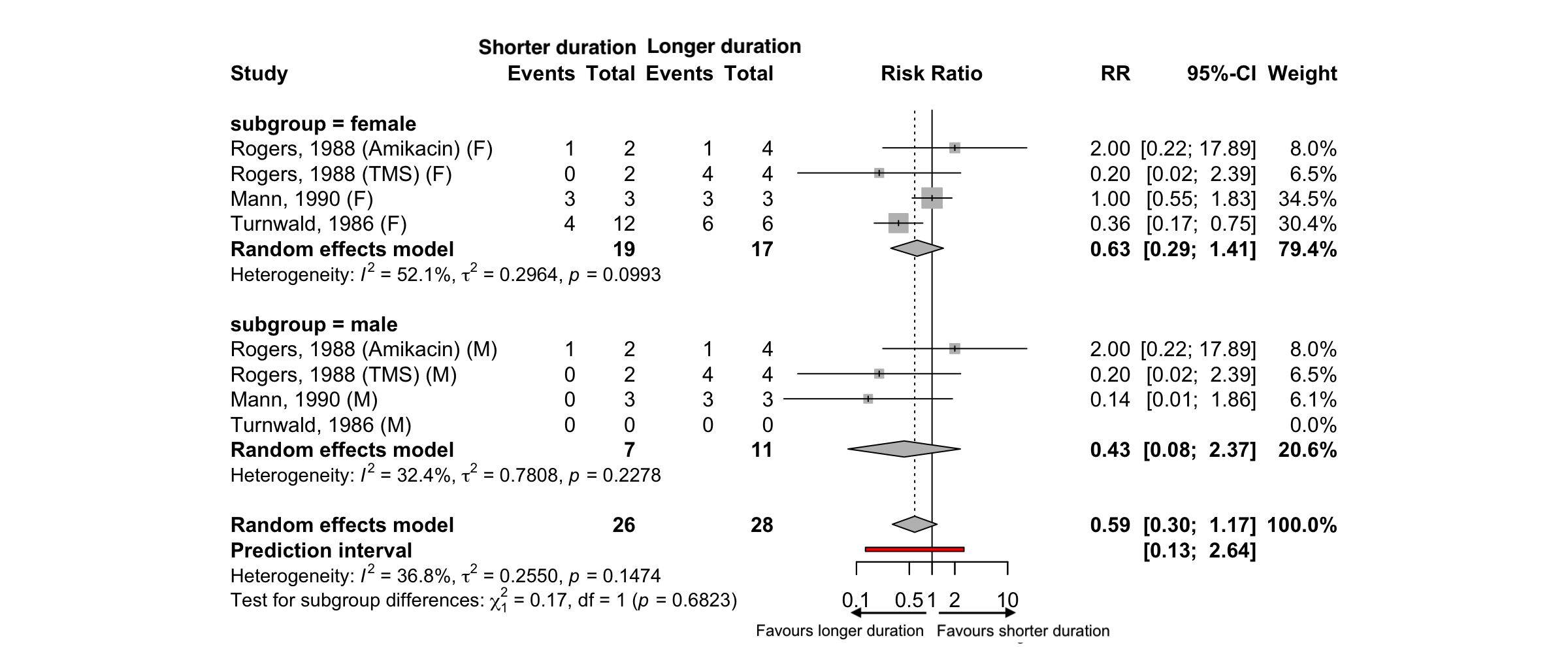
**Figure S4. Sub-group meta-analysis results of short versus long duration therapy microbiological cure rates for UTIs in dogs and cats by sex

**Appendix 6 - Sensitivity analyses**

**
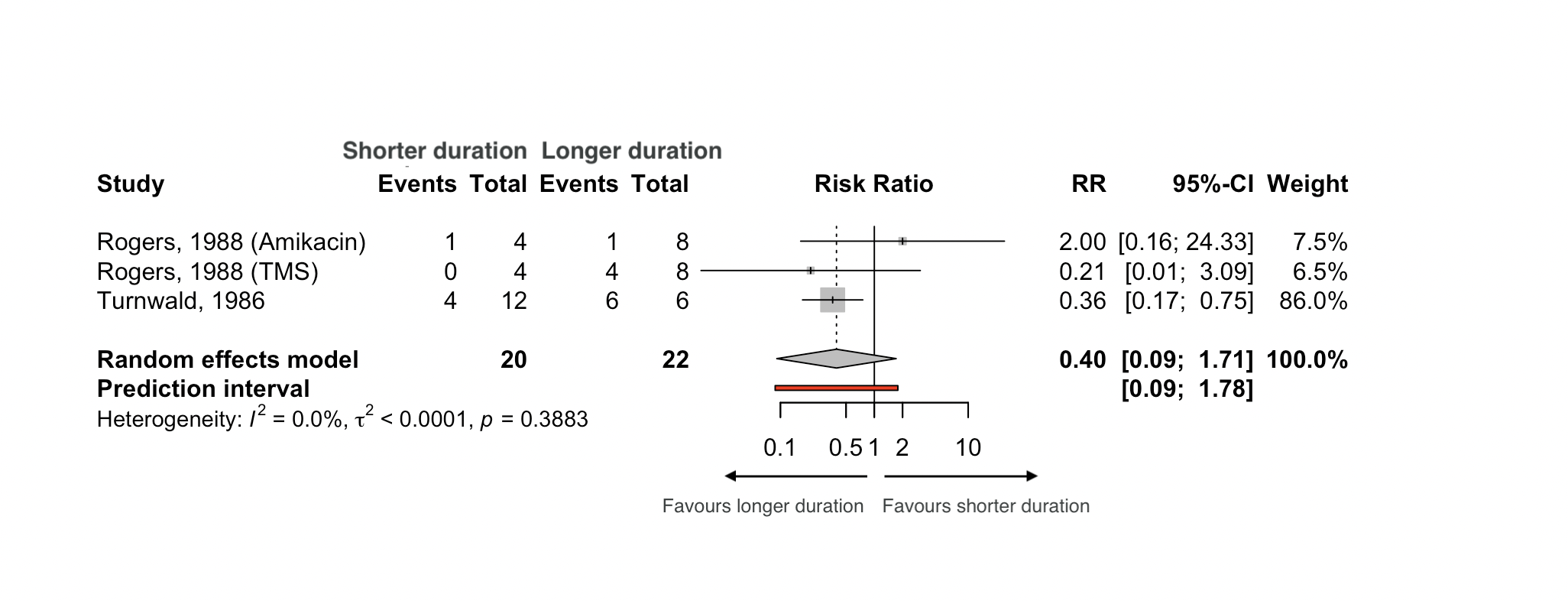
**Figure S5. Meta-analysis results of short versus long-duration therapy microbiological cure rates for UTIs in dogs and randomized controlled trials only.

**Appendix 7 – Funnel plot**

**
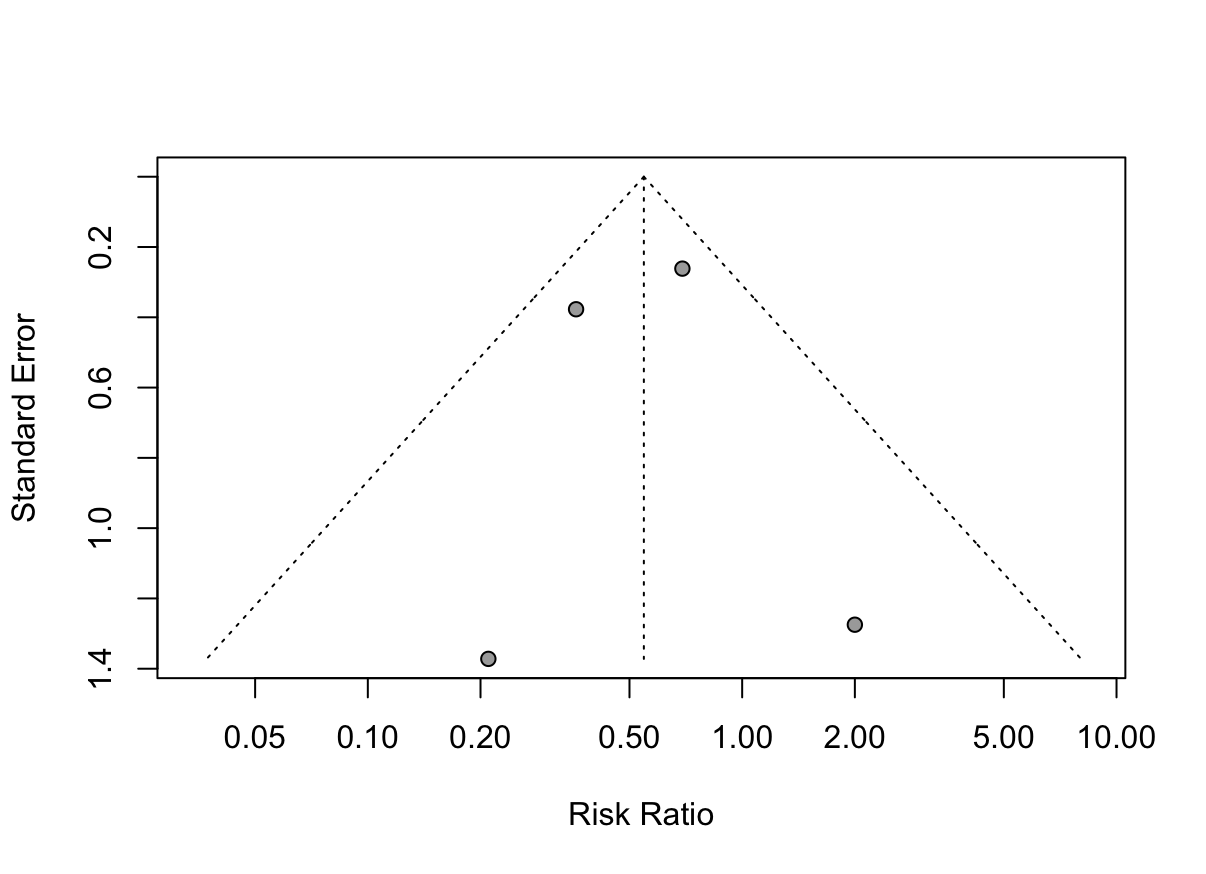
**

Figure S6. Funnel plot of effect size (RR) by standard error for studies included in the meta-analysis.
